# Fungal Contamination and Aflatoxin B1 Detected in Hay for Dairy Cows in South Italy

**DOI:** 10.3389/fnut.2021.704976

**Published:** 2021-09-21

**Authors:** Carlotta Ceniti, Nicola Costanzo, Anna Antonella Spina, Marinella Rodolfi, Bruno Tilocca, Cristian Piras, Domenico Britti, Valeria Maria Morittu

**Affiliations:** ^1^Department of Health Sciences University “Magna Græcia” of Catanzaro, Campus Universitario “Salvatore Venuta”, Catanzaro, Italy; ^2^Interdepartmental Services Centre of Veterinary for Human and Animal Health, Department of Health Science, Magna Græcia University, Catanzaro, Italy; ^3^Department of Earth and Environmental Sciences, University of Pavia, Pavia, Italy

**Keywords:** molds, mycotoxins, aflatoxin B1, food safety, food chain, dairy, feed, one health

## Abstract

The growth of filamentous fungi on fodder is recognized as responsible for fungal deterioration and mycotoxin contamination of the plant mass leads to economic losses in the dairy cow production system. Mycotoxin contamination has significant implications for human and animal health and is one of the major concerns in the food and feed chain. This research provides an insight into the variety of viable molds (i.e., filamentous microfungi) that can be isolated from hay produced in South Italy and destined to dairy cows. On different lots of hay (*n* = 55) collected from 20 dairy farms, a total of 33 different fungal species were identified. The most representative was *Cladosporium cladosporioides* (*n* = 46, 84%) followed by *Alternaria alternata* (*n* = 25, 45%), and *Rhizopus stolonifer* (*n* = 24, 44%). The species most closely related to aflatoxin (AF) contamination, *Aspergillus flavus*, was often isolated (*n* = 11, 20%). Regarding AF detection, all the hay samples were found to be scarcely contaminated by AFB1 and showed values from 0.0020 to 0.0077 mg/kg, below the limits established by European Union (EU legislation) (0.02 mg/kg). None of the samples were positive for *Aspergillia* and tested for AFB1 showed results exceeding established limits. Additionally, hay with moisture between 15.0 and 19.2% or crude ash on dry matter content ranging from 14.0 to 15.5% reported an increased presence of AFB1 (*p* < 0.05) compared to the other samples. All the analyzed hay samples, besides the presence of molds, can be considered safe for the presence of AFB1. Prevention of mold spoilage is mandatory to reduce the exposure of humans and animals to mycotoxins.

## Introduction

The occurrence of fungi or related mycotoxins in feed destined for animal production is one of the biggest concerns for animal and human health. The filamentous fungi found on hay belong to a wide range of fungal genera, which varies depending on many pre-harvest factors, such as the geographical location of the crop and weather conditions in the field (1, 2), the growing season (3), and agricultural practices, as well as the post-harvest factors, such as storage conditions (1, 4). It is known that the growth of filamentous fungi on fodder can cause problems related to the deterioration or contamination by mycotoxins. From this point of view, despite the mycological and mycotoxicological state of the forage, it is considered very important for risk assessment throughout the food chain (3, 5), there are few studies describing these aspects on hay (6).

Among mycotoxins, AFs represent the most important group; they are normally produced as toxic secondary metabolites mainly by two fungal species: *Aspergillus flavus* and *Aspergillus parasiticus* (7, 8). Aflatoxin B1 (AFB1), aflatoxin B2 (AFB2), aflatoxin G1 (AFG1), and aflatoxin G2 (AFG2) are the principal classes (9). Mycotoxins can easily enter the human food chain directly *via* plants products and indirectly *via* feed. Therefore, the occurrence of mycotoxin in feedstuff may be an important source of milk contamination and, thus, a serious hazard for human health. Aflatoxin M1 (AFM1) is a hydroxylated metabolite of AFB1 and approximately 0.5–6% of the ingested AFB1 is converted to AFM1 and secreted in milk in both humans and lactating animals (9). Several studies have reported a link between AFB1 and cancer occurrence and, according to the International Agency for Research on Cancer (IARC) (10), AFs are classified as group I or carcinogenic to humans. AFB1 is the most carcinogenic AF, and its presence in lactating animals feed could produce milk contaminated with AFM1, classified by the IARC as Group 2B, possibly carcinogenic to humans (11, 12). The susceptibility to AFs depends on several factors, such as age, the dose of secondary metabolites, the extent of exposure, gender, the species involved, and concomitant exposure to other hazardous toxins. The liver is the primary target organ in humans and animals, and the most common injury linked to AF exposure is hepatocellular carcinoma (HCC), although several other effects, such as reduction of immunological functions (13) are reported. Furthermore, AF contamination has a negative economic impact in terms of production loss by reducing both the animal feed intake and livestock productivity and reproductive capacity (14). To avoid the toxic effects due to AF presence, the European Union (EU) has stated maximum residue limits (MRL) for AFs both in the foods and feeds, as reported in the Regulation (EC) No 1881/2006 and its amending acts and, in the Directive 2002/32/EC (15) on undesirable substances in animal feed as amended by the Commission Regulation No 574/2011 (16), respectively. In the latter is indicated an AFB1 maximum load of 0.02 mg/kg for feed materials and a limit of 0.01 mg/kg for complementary and complete feeds, with the exception of compound feeds for some animal species. In particular, in compound feeds for dairy cattle, the AFB1 limit is set at 0.005 mg/kg. Less restrictive limits have been established in other countries, such as the United States, where it is established a threshold of 0.02 mg/kg in feeds and ingredients for dairy animals. The presence of mycotoxins in the feed has been widely investigated, however, the ratio between the concurrent presence of the potentially toxigenic species and of its mycotoxin still represents a critical issue deserving of attention. In this study, we report the results of mycological analysis of feed and relative occurrence of mycotoxin in feedstuff destined to the dairy animals collected in dairy farms located in the Calabria region, Italy. In addition, the influence of moisture and crude ash content of hay on AFB1 contamination was evaluated.

## Materials and Methods

**Figure 1:**
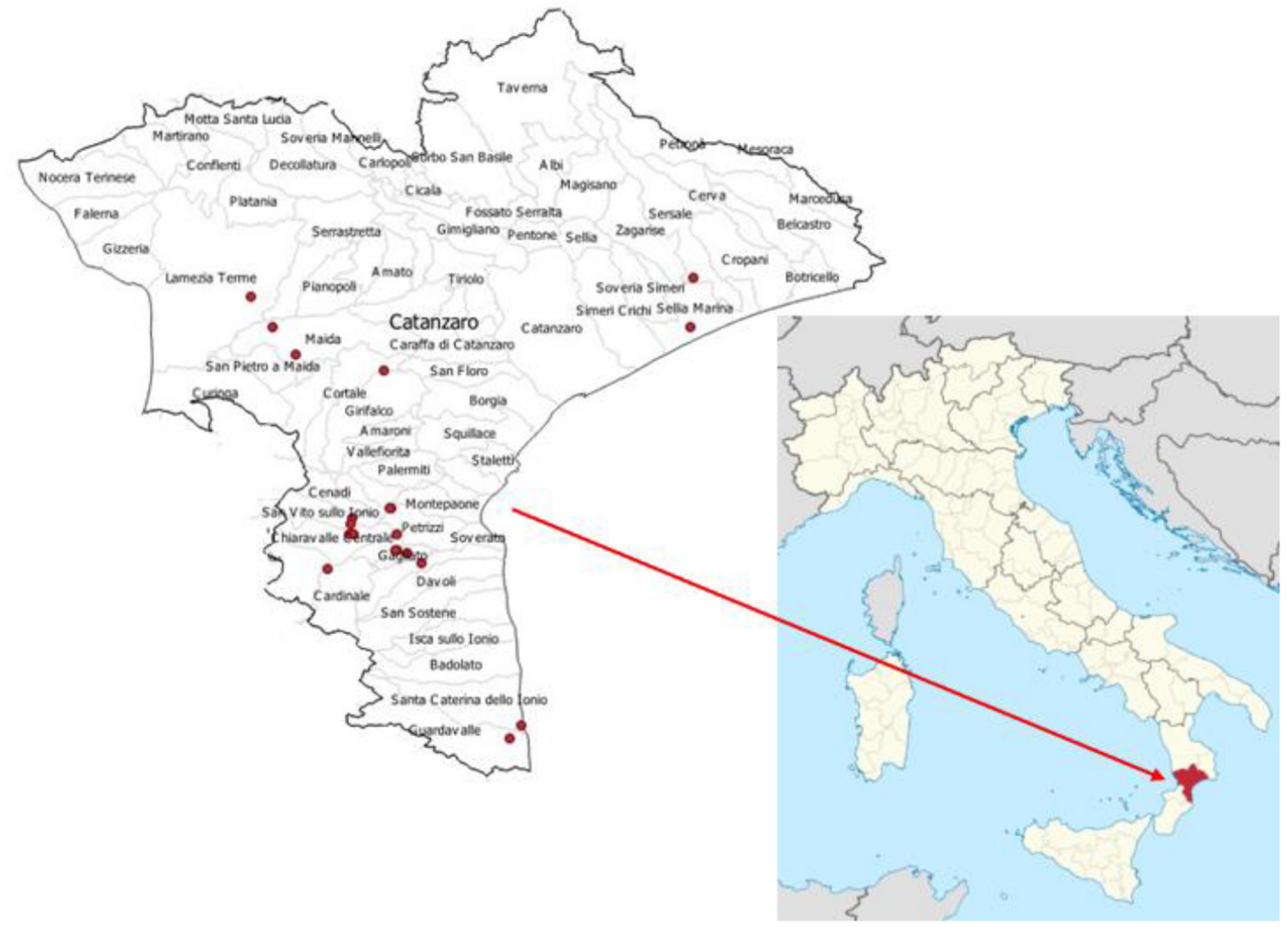
Map of the study area and sampling locations. The lower right image shows the Italian peninsula and the Calabria region (in red). The image on the left, on the other hand, shows the places (red dots) where the farms are located, and the hay samples were taken.

This study was carried out over a 3-month autumn period, from October to December. The research activity involved 20 dairy farms randomly chosen in the Catanzaro area (Italy) among the 29-producing hay. A map describing the study area and sampling locations is shown in Figure 1. Before sampling, a questionnaire was answered by the farmers to identify how many lots of hay were stored and its characteristics, such as the botanical species and the date and site of harvesting. Further information on the agronomic data, size, number of hay bales, and the storage type was acquired.

### Hay Sampling

The sampling criteria and hay sampling methods adopted for this research are those indicated in Annexes I and II of Regulation (EU) no. 691/2013, which amended Regulation (EC) no. 152/2009 concerning sampling and analysis methods for official feed controls. Briefly, for each lot, an aggregate sample of about 1 kg was obtained by sampling a minimum of 15 bales per lot randomly chosen, using a motorized corer (length 60.0 cm, internal diameter 22 mm). The sampling devices were thoroughly sterilized before sampling a new lot of hay. After collection, the aggregate samples were transported at room temperature to the laboratory and were carefully mixed and separated in two aliquots of 700 and 300 g for chemical and microbiological-toxicological analysis, respectively. In this latter case, the samples were stored at −20°C prior to the subsampling for the conventional mycological and mycotoxin analyses.

### Moisture and Crude Ash Determination

Moisture and crude ash content of the hay samples were determined as described by AOAC (Presidential Task Force on Best Practices for Microbiological Methodology) procedures (17). Briefly, the samples were ground by a Retsch SM 100 cutting mill equipped with a bottom sieve with an aperture size of 1.0 mm (Retsch GmbH, 42781 Haan, Germany), then, 5 g of hay were dried using the oven at 103°C for 4 h for the moisture content determination. The ash content was determined by muffle-furnace incineration of 5 g of the sample at 550°C for 3 h. The duplicate analyses were performed on each sample.

### AFB1 Determination

Determination of AFB1 in feed was conducted using the “Afla B_1_” ELISA kit (Tecna S.r.l., Italy) that has detection limits ranging from 1 to 40 ppb equivalent to 0.001–0.040 mg/kg. According to manufacturing instruction, specificity was 100% for AfB1, 5% ± 1 for AfB2, 19% for AfG1, and <1 for the AfG2. The relative SD of reproducibility was 6%. A subsample of 5.0 ± 0.1 g was used for the analysis: 25 ml of a 70:30 MeOH and H_2_O solution were added to the sample and then, filtered through Whatman 1 h before analysis. Briefly, 50 μl of the methanolic extract, dilution, or standard was mixed with 100 μl of conjugate directly in the dilution microliter wells. A 100 μl aliquot of this mixture was added to antibody-linked wells and incubated for 10 min at room temperature. Finally, 100 ml of stop solution was added and was left to stand for 5 min at standard temperature and pressure. The absorbance measurements were performed immediately at the wavelength of 450 nm using a SynergyTM Biotek HT microplate reader and the Gen 5TM software (BioTek Instruments Inc., Winooski, VT, USA).

### Mycological Analysis

The mycological investigation focused on the taxonomical identification of filamentous microfungi and was carried out by means of the moist chamber (MC) method. For each sample, 50 g of hay has been briefly washed under running tap water (30 s) to remove the extraneous propagules, then, drained and aseptically cut into fragments of about 1 cm length. The fragments were placed onto 150 mm Petri dishes containing a layer of Tap Water Agar (TWA, substratum composition: microbiological agar 15 g, tap water 1,000 ml). This methodological approach, providing only humidity, is particularly useful to stimulate the sporulation of fungi (development of both the asexual and sexual fruiting structures and spores). In particular, it promotes the growth of both the fungal pathogens, which use the plant material as a food base and the fungal saprophytes (18, 19). The dishes were kept at room temperature in natural day/night conditions for 14 days and constantly observed by means of a stereomicroscope (x5). From the third day, different microfungal fruiting structures emerged from vegetal tissues. Most of them, checked at high magnification (40–x 100), were directly identified at the genus level. All the different colonies were isolated into tubes containing the generic for fungi PDA medium (Potato Dextrose Agar, Sigma-Aldrich, final pH 5.6 ± 0.2), then, adequately transferred and manipulated for their complete identification, according to the related taxonomical keys (20–24). Only for the strains belonging to *Penicillium* and *Trichoderma* genera, identification was limited to the genus level (molecular approaches are required to obtain a fully reliable characterization of this kind of taxa). Moreover, although yeasts were not the object of the study, some strains have been detected from various samples; their presence has been however reported, with the meaning of delineating the total microfungal (both filamentous and unicellular) colonization of hay. Finally, taxa were discussed based on their origin and their toxigenic or pathogenic potential.

## Statistical Analysis

Mann–Witney test of GraphPad Prism version 8.3.0 for Windows (GraphPad Software, San Diego, CA, USA, www.graphpad.com) was used to compare the AFB1 levels of groups having different content of moisture and ash. A statistical significance was stated for *p* < 0.05.

## Results and Discussion

In this study, we report the first insight on the microfungal occurrence and AFB1 in hay collected in South Italy. Table 1 shows the moisture and crude ash values of the hay samples. The average moisture value was 12.21 ± 2.89%. The highest moisture content was 19.21% while the lowest was 7.27%. Although it is difficult to predict the moisture content for safe hay storage, Wittemberg et al. (25) reported that in alfalfa hay, moisture content of 17.5–21.6% is correlated with no mycelial development and very low sporulation. This would indicate a reduced risk even of the fungal metabolic activity in all the 55 samples considered, always taking into account that toxigenic molds can be strongly influenced by other modulating parameters, such as pH, temperature, and light during the storage (26). Gregory et al. (26) found that hays having 16% of moisture at bailing showed little heating and contained a small but diverse microbiota, resulting in good quality. On the contrary, the wetter hay bales contained many spore-forming bacteria but few fungi, surrounded by a layer of moldy hay on the surface (26). Other researchers (27, 28) have established an even stricter threshold for the moisture value of safe storage of hay, namely 15% as stated by Martinson et al. (28) for Orchardgrass hay storage with low microbial activity and absence of mold.

**Table 1:** Storage time, moisture, ash, and aflatoxin B1 (AFB1) of hay samples.

**Sample**	**Storage time (months)**	**Moisture (%)**	**Ash, on dry matter basis (%)**	**Aflatoxin B1, expressed on a moisture content of 12% (mg/kg)**
1	5	13.69	14.81	0.0038
2	5	12.04	12.30	0.0036
3	5	12.34	10.92	0.0031
4	5	8.72	10.64	0.0034
5	4	8.03	12.19	0.0031
6	2	10.30	12.72	0.0034
7	5	8.96	9.34	0.0026
8	5	10.12	15.49	0.0037
9	4	8.64	12.22	0.0028
10	4	9.78	12.15	0.0027
11	3	9.88	9.73	0.0031
12	2	9.74	9.84	0.0026
13	6	14.36	10.37	0.0077
14	7	15.52	10.08	0.0037
15	5	16.42	11.40	0.0034
16	4	7.48	10.18	0.0031
17	6	8.72	9.86	n.d.
18	6	7.64	9.80	n.d.
19	5	10.73	8.24	n.d.
20	5	11.95	8.92	0.0034
21	5	13.49	10.57	0.0025
22	5	10.93	11.73	0.0038
23	7	19.21	12.03	0.0037
24	6	17.42	14.15	0.0041
25	7	12.45	9.80	0.0029
26	2	14.42	12.63	0.0031
27	7	7.70	8.40	0.0024
28	7	7.77	8.91	0.0028
29	6	13.44	10.73	0.0027
30	6	11.58	8.09	0.0036
31	6	12.51	11.65	0.0023
32	5	13.07	7.37	0.0036
33	5	13.53	7.50	0.0021
34	6	12.86	9.41	0.0033
35	4	11.69	8.27	0.0026
36	3	18.02	9.91	0.0035
37	5	13.49	7.92	0.0023
38	6	7.27	9.54	0.0034
39	7	7.85	7.69	0.0055
40	6	9.74	6.28	0.0048
41	6	12.62	10.97	0.0038
42	6	13.47	9.40	0.0023
43	6	13.57	7.87	0.0025
44	6	13.54	8.66	0.0025
45	7	13.58	10.02	0.0035
46	7	14.04	10.97	0.0038
47	6	13.68	14.28	0.0035
48	5	15.29	12.01	0.0037
49	6	17.96	11.53	0.0031
50	6	13.47	7.98	0.0035
51	6	12.39	10.82	0.0033
52	7	13.05	11.22	0.0027
53	5	13.59	14.99	0.0029
54	4	14.73	14.94	0.0039
55	7	12.89	8.49	0.0020
Mean		12.21	10.51	0.0033
sd		2.89	2.14	0.0009
Max		19.21	15.49	0.0077
Min		7.27	6.28	0.0020

*The table summarizes the major features of the samples employed in this study. Parenthesis expresses the units for each column. Samples 17, 18, and 19 were not analyzed for the content of AFB1 and are marked as n.d*.

The moisture values of the hay samples in this study fully comply with this threshold value, except for seven samples, having moisture content above 15% (sample *n* 14, 15, 23, 24, 36, 48, and 49 in Table 1). Although, as discussed below, none of the hay samples exceeded the AFB1 tolerated content in the samples with high moisture content, it was found an AFB1 level (0.0036 ± 0.00031 mg/kg) higher (*p* = 0.0428) than in the hays having moisture <15% (0.0032 ± 0.00096 mg/kg).

For crude ash on dry matter, on the other hand, the average content is equal to 10.51% ± 2.14. In this case, the highest content was 15.49% and the lowest was 6.28%. The high content of crude ash is an indication of probable forage contamination with soil (29). In one study, it is reported that the values of ash above 14.0% on the dry matter for alfalfa hay are an indication of exogenous ash (29). Only six samples (refer to sample *n* 1, 8, 24, 47, 53, and 54 in Table 1), two of which from alfalfa, showed the ash values expressed on dry matter content higher than 14%. It is interesting to note that the AFB1 levels in this subset of samples (0.0037 ± 0.00042 mg/kg) were higher (*p* = 0.0229) than that observed on samples having ash content lower than 14% (0.0032 ± 0.00095 mg/kg), although all samples were below the legal limits. Indeed, as listed in Table 1, the values of AFB1 content ranged from 0.0020 to 0.0077 mg/kg with an average of 0.0033 mg/kg.

This study results showed that all the samples did not exceed the established limits of 0.02 mg/kg set for feed materials by Directive 2002/32/EC as amended by Commission Regulation (EU) 574/2011 (30) (Table 1). Many authors have reported concentrations that exceeded the established limits. Decastelli et al. (31) in northern Italy found that 8.1% of the feed samples analyzed were positive to confirmation analysis (AFB1), a value that was higher than the maximum allowable in the feed and the milk. In Iran, the concentration of AFB1 in the hay was higher (10%; 4/40) than the EU limit (32). Pleadin et al. (33) found 22.2% of AFB1-positive samples and detect that 12.3% of the feed samples had concentrations above the limit. In Tanzania, Mohammed et al. (34) found that AFB_1_ was present in 65% (13/20) of the feed samples and 61.53%, exceeding both the Tanzania Food and Drug Authority and European commission maximum limits of 5 ng/g (0.005 mg/kg) for complete dairy animal feed. Inversely, other studies have found a relatively high number of contaminated samples but rarely exceeding the established legal limit. In China, 42% of the samples contained AFB1 ranging from 0.05 to 3.53 μg/kg, below the legal limit in European and Chinese (10 μg/kg, equivalent to 0.01mg/kg) limits (35). In Portugal, Martins et al. (36) in a 10-year study, found 37.4% positive samples with contamination ranging from 1 to 74 μg/kg, and only 6.2% of samples exceeded the maximum limit established in Portugal (5 μg/kg, equivalent to 0.005 mg/kg).

The climate conditions could influence mold spoilage and favorable environmental conditions are critical for the production of mycotoxin. An interesting study that evaluated the presence of mycotoxins in relation to the geographical area, reported that more than half of the specimens collected in Europe exceeded the legal limit of quantification; in the Asian-Pacific area, contaminations by AF were more common (37). Furthermore, the level of AFB1 in feed samples collected during the winter season was found to be higher than those collected in the summer months (38). Therefore, many explanatory variables may affect the probability of AFB1 production and worldwide scientific evidence indicate the complexity to predict the consequent actual risk for animals and humans.

The occurrence of mycotoxin in animal feed could results in the carry-over effect of AFM1 in milk at dairy farms. When dairy cattle eat feedstuffs containing AFB1, this toxin is metabolized in AFM1 and, thus, excreted in milk, representing the main concern for public health. In milk, the limit of AFM1 is 0.05 μg/kg (7). The low frequency of AFM1 contamination in milk is the consequence of control of AFM1 in milk joined with the routine surveillance of AFB1 in the primary production of feeds for dairy animals. In an interesting review, Min et al. (39) underlined the great risk related to high AFM1 concentrations in raw milk in several countries. The consumption of contaminated raw or pasteurized milk can be considered a significant risk, especially for the health of infants and children since milk is the major constituent of their diet (40). In Italy, it was found that no commercial sample exceeded the limit defined at the community level for AFM1 in milk (0.05 μg/kg) (41). A correlation between AFs in feed and AFM1in milk has not been detected always, as reported. Blanco (42) reported that although none of the feed samples examined exceeded the EU maximum content for AFB1 in feeding stuff for dairy animals, some bulk milk samples (*n* = 3) exceeded the maximum level for AFM1 in milk (50 ng/kg) established in EU. Monitoring of feedstuffs is a useful tool to try and minimize AF contamination of milk.

Concerning mold characterization, the results are synthesized in Table 2. A total of 33 different taxa were identified, mainly belonging to the Phylum Ascomycota and, within it, to the genera *Cladosporium, Alternaria, Aspergillus, Fusarium*, and *Epicoccum*. The Phylum Mucoromycota was represented by species belonging to the genera *Rhizopus* and *Mucor*.

**Table 2:** Mycological results of hay samples.

**Phylum**	**Genus**	**Species**	**No. isolates**	**No. isolates/No. total samples (%)**
*Ascomycota*	*Acremonium*	*A. strictum* W. Gams	3	5.45
	*Alternaria*	*A. alternata* (Fr.) Keissl.	25	45.45
	*Arthrinium*	*A. phaeospermum* (Corda) M.B. Ellis	1	1.82
	*Ascochyta*	*A. medicaginis* Qian Chen & Cai	4	7.27
	*Aspergillus* (section *Flavi*)	*A. flavus* Link	11	20.00
	*Aspergillus* (section *Fumigati*)	*A. fumigatus* Fresen.	2	3.64
	*Aspergillus* (section *Nigri*)	*A. niger* Tiegh.	16	29.09
	*Aspergillus* (section *Circumdati*)	*A. ochraceus* G. Wilh.	5	9.09
	*Aspergillus* (section *Versicolores*)	*A. sydowii* (Bainier and Sartory) Thom and Church	1	1.82
	*Aspergillus* (section *Terrei*)	*A. terreus* Thom	3	5.45
	*Aspergillus* (section *Versicolores*)	*A. versicolor* (Vuill.) Tirab.	6	10.91
	*Botrytis*	*B. cinerea* Pers.	2	3.64
	*Chaetomium*	*C. bostrychodes* Zopf	2	3.64
	*Cladosporium*	*C. cladosporioides* (Fresen) G.A. De Vries	46	83.64
	*Epicoccum*	*E. nigrum* Link	12	21.82
		*E. sorghinum* Aveskamp, Gruyter and Verkley	8	14.55
	*Eurotium*	*Eurotium chevalieri* L. Mangin	8	14.55
	*Fusarium*	*F. graminearum* Schwabe	3	5.45
		*F. oxysporum* Schltdl	7	12.73
		*F. verticillioides* (Sacc.) Nirenberg	11	20.00
	*Nigrospora*	*N. oryzae* (Berk and Broome) Petch	8	14.55
	*Penicillium*	*P. purpurogenum* Stoll	5	9.09
		*P. simplicissimum* (Ouden) Thom	7	12.73
		*Penicillium* sp.	9	16.36
	*Pseudopithomyces*	*Pseudopithomyces chartarum* (Berk and Curtis) Li, Ariyaw and Hyde	2	3.64
	*Sordaria*	*S. fimicola* (Roberge ex Desm.) Ces. and De Not.	2	3.64
	*Stachybotrys*	*S. chartarum* (Ehrenb.) S. Hughes	2	3.64
	*Torula*	*T. herbarum* (Pers.) Link	1	1.82
	*Trichoderma*	*Trichoderma* sp.	3	5.45
*Asco- Basidio-mycota*	Yeasts	*-*	27	49.09
*Mucoromycota*	*Mucor*	*M. circinelloides* Tiegh.	1	1.82
		*M. plumbeus* Bonord.	14	25.45
		*M. hiemalis* Wehmer	2	3.64
	*Rhizopus*	*R. stolonifer* (Ehrenb.) Vuill.	24	43.64

The highest percentage of sample was colonized by *Cladosporium cladosporioides* (*n* = 46, 84%) followed by *Rhizopus stolonifer* (*n* = 35, 64%) and *Alternaria alternata* (*n* = 25, 45%). *Cladosporium* is one of the most frequent airborne species, mainly found in the inside and outside environments of agricultural context and dairy farms, responsible for damage of sheep and cow cheese surface (forming black tight spots); moreover, *Cladosporium* spores are known to play a role as aeroallergens and the prolonged exposure to high spore concentrations could cause upper respiratory symptoms related to chronic allergy and asthma (43, 44). *Rhizopus stolonifer* is a typically atoxigenic post-harvest mold of fruit and vegetable, and mold spoilage occurs mainly during storage, transport, and commercialization (45). *Alternaria alternata*, which was rescued in a percentage of 45%, is a ubiquitous, saprophytic fungus isolated in a variety of habitats, commonly in dead plant materials, and is also a plant pathogen causing disease on several crops (46, 47). The genus *Alternaria* includes some species related to the production of toxins, such as alternariol, altenuene, tenuazonic acid, and altertoxin, thus implying a serious hazard (48). In an interesting study, spices and herbs marketed in Lebanon were analyzed for *Alternaria* mycotoxins and found a high incidence (89%) of sample contamination, highlighting the high susceptibility of these matrices to these potential harmful mycotoxins (49).

Considering the purposes of this study, the result of *A. flavus* as the most frequent toxigenic species isolated (*n* = 11, 20%), deserves to be highlighted. It is the main source of AFB1, of which the ubiquitous growth can occur at any point in the pre- or post-harvest stage, making it difficult to control the contamination (13). The dairy feed may represent suitable media for the growth and proliferation of *Aspergillia*. In a survey conducted in Nigeria, on 144 feed samples destined to dairy herds, 55.8% of the isolates *Aspergillia* were identified as *A. flavus* but only 12 (25.0%) were identified as aflatoxigenic strains (50). In Iran on 110 samples collected in dairy farms, the most frequent isolated fungi (*Aspergillus fumigatus, A. flavus, Aspergillus niger, A. parasiticus*, and *Aspergillus oryzae*) were found in the hay samples (92%) (51). Ghisian et al. (52), in Iran during the winter season, found that predominant fungi isolated were *Aspergillus* species (37.4%) followed by *Penicillium* (23.7%), *Fusarium* (17.5%), *Cladosporium* (9.1%), *Alternaria* (4.3%), *Rhizopus* (3.9%), and *Mucor* species (3.4%). A not negligible detection of *Penicillium* and *Fusarium* emerged in this study too, and some data (i.e., *Fusarium verticillioides, n* = 11, 20%; *Fusarium oxysporum, n* = 7, 13%) could deserve future insights, still concerning a potential toxigenic risk.

Interestingly, in the current survey, none of the samples colonized by *Aspergillia* exceed the limit of AFB1, thus indicating that the presence in the hay of detectable fungal propagules not necessarily implies the production—and the risk—of mycotoxins. In a study performed by Granados-Chinchilla et al. (53) on 1,200 rice samples, collected from 20 states across India, was found that all the samples showed the presence of *Aspergillia*, predominantly represented by *A*. *flavus* (*n* = 1002, 67.8%); these samples were positive to AFB1 but only 2% showed contamination above the permissible limits (>30 μg/kg) (assessed by Elisa). These findings are in agreement with Udom (54) that showed a low incidence of aflatoxigenic *A. flavus* and high incidence of AFB1, probably due to rapid depletion of the vegetative phase of the organism due to harsh environmental conditions with no significant consequence on thermostable AF. Similarly, Omeiza et al. (50), in a study carried out in 2018, reported a high percentage of positive samples for AFB1 associated with a low incident rate of aflatoxigenic strains of *A. flavus*. International data state that AF contamination of feedstuff can both lead to considerable production losses and be a great hazard for animal and human health. The current study confirmed that: hay used as feed materials for dairy cattle are naturally colonized with filamentous fungi and yeasts, often occurring in the field because of the infection of plant symbiotic fungi as phytopathogens; in well-preserved forages, the metabolic activity of molds can be greatly reduced, making it impossible to set a direct and replicable ratio between the presence of a certain species and its mycotoxin; the hay-making process, as well as the post-harvest handling and storage of these organic products, are key conditions for preventing their rapid spoilage and, consequently, for guaranteeing quality and safety to the whole productive chain. The results of this study agree with those obtained from similar studies, and confirm that random inspections of hay, by means of microbiological and/or biochemical techniques, are recommended and should be constantly performed. Especially, frequent AFB1 monitoring should be established, particularly, in hays with high moisture and crude ash content, to prevent the introduction of this major toxin into the food chain. We conclude that the quality of raw materials and good practices of storage are essential for the prevention of spoilage and mycotoxin spread and, thus, for human and animal health. Finally, since this toxigenic matter may vary according to the geographical area and climatic features, we propose this first analysis performed in Calabria as a reliable starting point for a more accurate and proven strategy of territorial assessment.

## Data Availability Statement

The raw data supporting the conclusions of this article will be made available by the authors, without undue reservation.

## Author Contributions

NC and CC contributed to the conception and design of the study. VM, CC, and MR contributed to methodology. AS organized the database. VM performed the statistical analysis. VM and AS performed the validation. CC, AS, and MR performed the analysis. MR performed the mycological investigation. VM contributed to data curation. CC and NC wrote the first draft of the manuscript. BT and CP wrote sections of the manuscript. NC, VM, and DB contributed to supervision of the project. VM and DB contributed to resources acquisition. All authors have read and contributed to writing, editing the final version of the manuscript, and agreed to the published version of the manuscript.

## Conflict of Interest

The authors declare that the research was conducted in the absence of any commercial or financial relationships that could be construed as a potential conflict of interest.

## Publisher's Note

All claims expressed in this article are solely those of the authors and do not necessarily represent those of their affiliated organizations, or those of the publisher, the editors and the reviewers. Any product that may be evaluated in this article, or claim that may be made by its manufacturer, is not guaranteed or endorsed by the publisher.
